# Acute Effects of High-Load Training to Failure vs. Non-Failure on Posture and Core Endurance in Collegiate Weightlifters: A Crossover Study

**DOI:** 10.3390/jcm15082815

**Published:** 2026-04-08

**Authors:** Osama R. Abdelraouf, Amr A. Abdel-Aziem, Nouf H. Alkhamees, Zizi M. Ibrahim, Ehab M. Aboelela, Reem S. Dawood, Ahmed A. Ashour

**Affiliations:** 1 Physical Therapy Program, Batterjee Medical College, Jeddah 21442, Saudi Arabia; pt4.jed@bmc.edu.sa (O.R.A.); pt9.jed@bmc.edu.sa (R.S.D.); 2 Department of Biomechanics, Faculty of Physical Therapy, Cairo University, Giza 12611, Egypt; 3 Department of Physical Therapy, College of Applied Medical Science, Taif University, Taif 21944, Saudi Arabia; amralmaz@tu.edu.sa; 4 Department of Rehabilitation Sciences, College of Health and Rehabilitation Sciences, Princess Nourah bint Abdulrahman University, P.O. Box 84428, Riyadh 11671, Saudi Arabia; zmibrahim@pnu.edu.sa; 5 Fitness Pioneers Institute, Jeddah 23451, Saudi Arabia; ehabpt6@gmail.com; 6 Biomechanics Department, Faculty of Physical Therapy, October 6 University, Giza 12585, Egypt; ahmed.ashour.pt@o6u.edu.eg

**Keywords:** core endurance, high-load training, muscle failure, upper extremity, collegiate weightlifters

## Abstract

**Background:** Weightlifters commonly use upper-extremity high-load training, which encompasses techniques ranging from momentary failure to non-failure. However, little is known about how this training affects posture and core endurance, despite knowing that these factors are risk factors for weightlifting injuries. Therefore, this study aimed to determine the immediate effects of upper-extremity high-load training to momentary failure versus non-failure, using the dumbbell overhead press, on posture and core endurance in recreational collegiate weightlifters. **Methods:** Fifty recreational weightlifters aged 18–24 with two years of upper extremity resistance training experience were recruited for this study. The participants performed dumbbell overhead press exercises under high-load failure (HL-F) and high-load non-failure (HL-NF) conditions two days after 1RM testing and calculation of the 80% 1RM load. The study analyzed postural changes using photographic data processed in Kinovea, while core endurance was assessed during a prone plank test. Standardized warm-ups, controlled exercise execution, and pre- and post-exercise assessments were conducted to measure core endurance and postural alterations. **Results:** The thoracic kyphosis angle, together with scapular balance angle and lateral scapular slide distance, increased significantly after HL-F compared to the unloading state, while the craniovertebral angle and prone plank time decreased significantly (*p* < 0.05). The HL-NF condition showed no statistically significant differences relative to the unloading measurements (*p* > 0.05). The unloading measurements across testing days were consistent, indicating no carryover effect (*p* > 0.05). **Conclusions:** The findings indicate that high-load training to failure adversely affects posture and core endurance, increasing fatigue and potentially increasing the risk of acute injuries. Non-failure training maintains stability, underscoring the importance of strategic program design for achieving optimal performance while minimizing adverse effects.

## 1. Introduction

Weightlifting is a widely practiced activity aimed at increasing strength and achieving muscle hypertrophy through repeated muscle actions against progressive resistance, exceeding the demands of daily activities [1]. In recent years, weightlifting has gained substantial popularity as a recreational activity, with college students representing nearly 35% of participants [2,3]. For those young adults, weightlifting improves both physical appearance and mental well-being. It boosts self-esteem, fosters a sense of control, and helps counter societal pressures, promoting a healthier and more confident self-image [4].

Despite its benefits, weightlifting carries a significant risk of injury, particularly when performed incorrectly [2]. Over a six-year period, the annual incidence of weightlifting-related injuries increased from 86,910 to 109,961, with nearly 40% involving the shoulder and upper trunk [5]. Common risk factors include sudden weight drops due to loss of balance and improper technique linked to poor body alignment [6]. These injuries underscore the importance of proper form and core stability during weightlifting. The core musculature is essential for stabilizing the trunk and maintaining proper posture during dynamic movements, playing a critical role in power generation and enhancing overall movement efficiency [7].

Research has extensively explored key training variables, such as load intensity, repetitions, and sets, which collectively define training volume [8,9,10]. Traditional weight training typically associates strength gains with high-load (HL) lifting, but previous studies have shown inconsistencies in defining HL based on one-repetition maximum (1RM) percentages. Early studies defined high-load (HL) training as loads exceeding 60% [11] or 70% [12] of the one-repetition maximum (1RM). However, a recent systematic review and meta-analysis established a more precise criterion, defining HL as ≥80% of the 1RM [13].

Moreover, a growing belief in weightlifting suggests that training into momentary muscle failure optimizes neuromuscular gains when a muscle cannot complete another concentric repetition. This principle has driven much of the earlier research on training to failure [14,15,16]. However, recent studies challenge this notion, demonstrating that training to failure is comparable to non-failure training for enhancing muscle hypertrophy, strength, and architecture [17,18]. These findings suggest that alternative approaches may be equally effective without the added strain of pushing to failure.

In weightlifting sports, pressing motions such as an overhead press are key for engaging the upper quarter muscles [19]. The overhead press, also known as the shoulder or military press, is a compound exercise that involves the hands, arms, and shoulder girdle muscles [20]. Given the importance of core stabilization in preventing injuries, pressing exercises, particularly the dumbbell overhead press, are known for their high stabilization demands [21,22].

Despite extensive research on load intensity and training to failure in relation to strength, motor unit recruitment, and hypertrophy, no prior studies have explored its effects on body alignment and core endurance. Addressing this gap is essential, as it provides valuable insights for recreational lifters seeking to optimize training strategies, enhance performance, and reduce injury risk. Therefore, this study investigated the acute effects of upper-extremity high-load training to failure (HL-F) versus non-failure (HL-NF) using the dumbbell overhead press on posture and core endurance in collegiate weightlifters.

## 2. Materials and Methods

### 2.1. Study Design

This within-subject crossover study was conducted in the college physiotherapy lab between February 2024 and October 2024. The ethical committee of Batterjee Medical College (RES-2024-0217) reviewed and approved the study procedures in compliance with the most recent version of the Declaration of Helsinki. All participants provided written informed consent after receiving a detailed explanation of the study objectives and their right to withdraw without consequences.

### 2.2. Sample Size

G*POWER statistical software (version 3.1.9.3; Universität Düsseldorf, Düsseldorf, Germany) was utilized to determine the required sample size for the study. A total sample size of 45 weightlifters was accepted based on an alpha of 0.05, a power of 80%, and a medium effect size of 0.50 [18]. An unpublished pilot study on 12 weightlifters further supported the reported effect size.

### 2.3. Participants

This study included 50 recreational weightlifters (28 males, 22 females; body mass: 84.2 ± 8.1 kg; height: 175.3 ± 4.6 cm; BMI: 26.8 ± 1.4 kg/m^2^) aged 18–24 years. These participants were recruited through hanging posters, distributing flyers, and sending mass emails. To qualify as resistance-trained for the study, participants must engage in upper extremity resistance weight training twice a week, at an intensity that reaches 80% of the 1RM, with an average of four sets per training session and seven to nine repetitions. The lifting experience should be at least two years before the start of the study [23].

Weightlifters who used hormonal supplements, participated in professional powerlifting or bodybuilding events, had shoulder pain, previous musculoskeletal injuries, or missed more than three weeks of lifting workouts over the last six months were excluded [3]. All inclusion and exclusion criteria were assessed through a standardized screening questionnaire and personal interview conducted by trained research assistants. A total of 62 weightlifters were initially assessed for eligibility; however, 12 were excluded for failing to meet the specified inclusion criteria.

**Figure 1:**
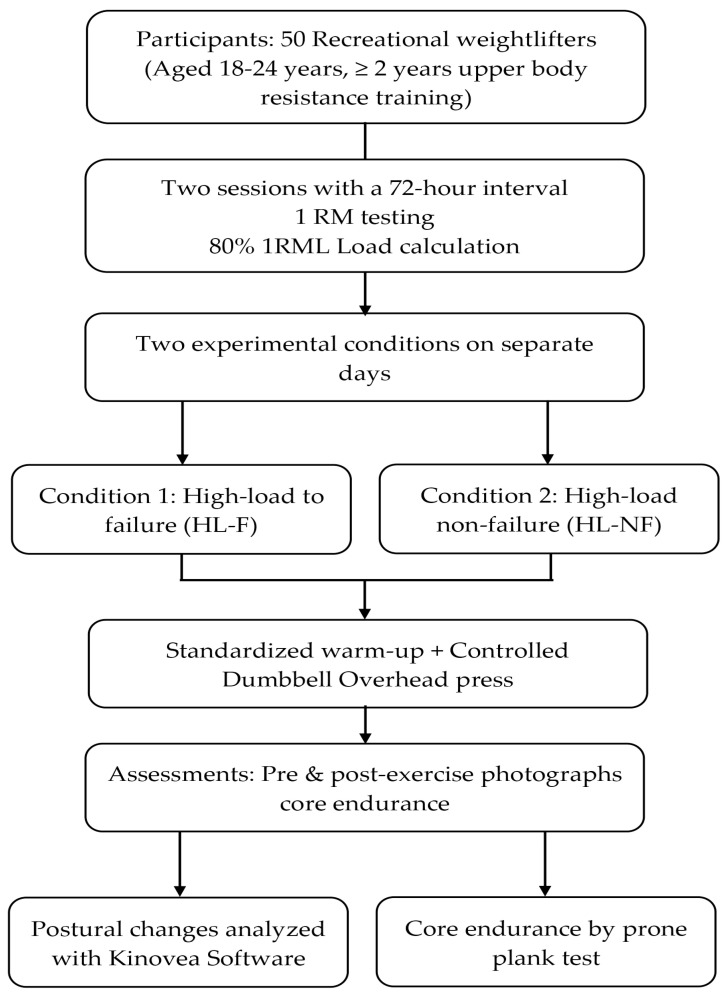
The study flowchart.

A within-subject crossover design was used to include all participants in a single group to maximize homogeneity and reduce between-subject variances resulting from physical, strength, and training level characteristics [24]. The study flowchart is shown on Figure 1.

### 2.4. Procedures

#### 2.4.1. One-Repetition Maximum (1RM) Testing

At first, the weightlifter attended the college physiotherapy lab for 1RM familiarization and determination. The testing procedure was by the National Strength and Conditioning Association recommendations [25]. The session began with 10 min of low-intensity general and specific warm-up exercises. These included 5 min of treadmill walking at a normal pace, clockwise and counterclockwise arm circular movements, and overhead press with light weight.

The weightlifter was then given a detailed explanation of correctly performing the overhead press exercise. Participants were instructed to stand with their feet positioned shoulder-width apart, holding dumbbells at shoulder level with their palms facing forward and elbows positioned slightly ahead of the torso. They were told to engage the core, keep the neutral spine, and press the weight overhead in a straight line until the arms were fully extended without locking the elbows. The emphasis was on preventing excessive lower back arching by keeping the ribcage down and the gluteus and core active for stability. They were also told to lower the weight in a controlled manner to the starting position while keeping their wrists in line with their forearms. This instruction form was adopted from the NCSA manual guidelines [26].

The researcher observed the exercise form during the testing trial, which ended when the performance technique was deviated. The first lifting load was set according to the weight that each participant stated as their failure point after one repetition. This first weight was then gradually increased until the actual 1RM was obtained, and no more than five trials were done daily. A two-minute rest interval was set between the tests to ensure that the results of the tests would not be affected by fatigue [27].

The 1RM was retested after 72 h to ensure reliability. If the difference between the first and second tests was more than 5%, the procedures were repeated after another 72 h [13,15]. Only 12 participants required three trials to determine the 1RM. For each participant, 80% of the 1RM was calculated and recorded to be used in the high-load testing condition [13].

#### 2.4.2. Testing Protocol

Before the testing session, participants were informed about the criteria for momentary muscular failure, defined as the point at which they could no longer perform an additional repetition with correct technique. In contrast, during training to non-failure, participants voluntarily stopped their repetitions based on their perceived fatigue, halting three to four repetitions (Reps in Reserve) short of reaching the point of muscular failure [18].

In addition to the day designated for 1RM determination, each weightlifter participated on two testing days, depending on whether failure was reached. A 72 h recovery period was provided between testing days to prevent fatigue or carryover effects [13]. Testing was conducted at the same time of day to minimize circadian effects, and the order of days was randomized using a random number generator. Every testing day typically began with warming up similar to the routine used during the determination of the 1RM, followed by capturing the overhead press exercise in its unloaded state, which involved performing the exercise without holding any weight [28].

The number of sets performed before measuring the outcomes depended on each participant’s usual lateral raise training routine. This personalized approach was selected to replicate real training conditions. Typically, participants completed 3–5 sets (mean = 4.1 ± 0.8 sets) of overhead press at 80% 1RM, with 2 min rest intervals, prior to post-exercise assessments [29].

### 2.5. Outcome Measures

#### 2.5.1. Photographic Postural Analysis

Two digital cameras (Canon Inc., Tokyo, Japan, model EOS 750D) were used to capture lateral and posterior views of each participant. Photographic analysis of the upper quarter posture from sagittal and posteroanterior perspectives was conducted.

Postural analysis was conducted using Kinovea software (version 0.9.5), which is a valid and reliable tool that can measure accurately at distances of up to 5 m from the subject [30]. Participants were instructed to assume and maintain their regular standing posture typically used during exercise routines, to ensure consistency in biomechanical alignment. The camera, placed on a tripod, was positioned 2 m away from the participants, with its height adjusted to align with the external auditory meatus in the sagittal plane and the skull’s greatest protrusion in the frontal plane [31]. The final set of exercises on each day involved recording a video and analyzing the image of the final set repetition. Independent physical therapists, blinded to the study purpose, performed photographic analyses. These therapists were well-trained and had prior experience in conducting photographic postural analysis, ensuring interrater reliability and consistency in the measurements.

**Figure 2:**
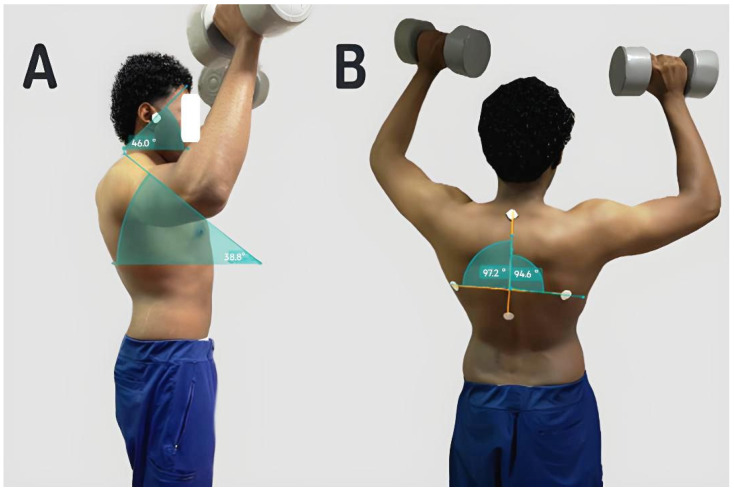
Measurement of craniovertebral angle, thoracic kyphosis angle (**A**), scapular balance angle, and the lateral scapular shift distance (**B**).

Four measurements, two from each recording plane, were analyzed as follows: the craniovertebral angle and the thoracic kyphosis angle were measured from the sagittal plane. The craniovertebral angle is formed by the intersection of a line drawn from the tragus of the ear to the horizontal line through the spinous process of C7. The thoracic kyphosis angle is formed at the intersection of two lines passing through T12 and C7 (sagittal view—Figure 2A) [32]. A craniovertebral angle (CVA) of less than 48 degrees is defined as forward head posture, whereas a thoracic kyphosis angle greater than 40 degrees is considered hyperkyphosis [33].

Moreover, from the anteroposterior view, the scapular balance angle was calculated by determining the angular difference between a line connecting the inferior angles of both scapulae and a vertical reference line aligned with the spinal column [23]. Additionally, the lateral scapular shift distance was measured from the scapulae’s inferior angle to the thoracic vertebrae’s spinous process at the same level (Figure 2B) [34]. Asymmetry in the position of the scapulae or scapular dyskinesis is defined as a difference between the two sides of the body of more than 7 degrees in the scapular angle or more than 1.5 cm in the lateral shift distance [35]. Postural analysis used a single photographic frame from the final repetition of the final set, representing maximal fatigue, and was analyzed by independent, blinded therapists using Kinovea software (v.0.9.5). No averaging across repetitions was conducted.

#### 2.5.2. Prone Plank Time

The endurance prone plank test is widely recognized as a valid, reliable, and practical tool for assessing overall core muscle endurance, especially in athletes who have had at least one familiarization session beforehand [36,37]. To begin the test, participants got into the standard plank position, lying face down, supported on their forearms and toes. Their elbows were aligned directly under their shoulders, with forearms and hands extended forward. The test assessor instructed participants to maintain a straight body from head to heels. The test was ended immediately when participants could not maintain the neutral plank position despite verbal cueing (a straight head-to-heels posture without pelvic sagging, hip piking, or repositioning of the forearms or feet), and at that point, the time was recorded as plank endurance [38].

### 2.6. Statistical Analysis

The data were analyzed using the Statistical Package for Social Sciences version 20 (SPSS Inc., Chicago, IL, USA). The data were tested for normality and homogeneity of variance assumptions before the final analysis. The data’s homogeneity and normal distribution were confirmed by the Shapiro–Wilk test and Levene’s test (*p* > 0.05). This exploration was conducted as a prerequisite for parametric variance analysis. Repeated-measures ANOVA was used to assess the effect of upper-extremity HL-F versus HL-NF training on upper-body quarter posture and core endurance. A Bonferroni-adjusted post hoc test was used to identify the source of significant differences, providing a more conservative control of the family-wise Type I error rate. The significance level was set at 0.05 for all statistical tests.

## 3. Results

Through HL-F training, the value of CVA and prone plank time during unloading conditions was significantly greater than those during the loading condition (*p* = 0.003 and 0.002, respectively). In contrast, the values of the thoracic kyphosis angle, scapular balance angle, and lateral scapular slide distance during the unloading condition were significantly lower than those during the loaded condition (*p* = 0.001). Concerning HL-NF training, there were no significant differences between the unloading and loading conditions values of the CVA, thoracic kyphosis angle, scapular balance angle, lateral scapular slide distance, and prone plank time (*p* = 0.341, 0.272, 0.068, 0.164, 0.182, respectively).

During unloading conditions, there was no significant difference between the values of the HL-F and HL-NF of the CVA, thoracic kyphosis angle, the scapular balance angle, the lateral scapular slide distance, and the prone plank time (*p* = 0.459, 0.658, 0.510, 0.815, 0.645, respectively). However, the CVA and prone plank time values during HL-F training were significantly lower during loading conditions than in HL-NF training (*p* = 0.005, 0.015, respectively). In addition, the values of the thoracic kyphosis angle, scapular balance angle, and lateral scapular slide distance during HL-F training were significantly greater than those during HL-NF training (*p* = 0.025, 0.001, 0.001, respectively), as shown in Table 1.

## 4. Discussion

This research study contributes new findings to weightlifting science by evaluating the effects of HL-F training versus HL-NF training on posture and core endurance. It provides preliminary implications for injury prevention and performance. Postural alignment and core endurance show negative alterations following HL-F training, yet HL-NF training sustains stable performance.

### 4.1. Effect of HL-F on Upper Quarter Posture

When weightlifters trained until failure under heavy loads, their CVA value decreased, and their thoracic kyphosis angle increased substantially, suggesting an increase in forward head posture (FHP) due to fatigue of cervical and upper thoracic stabilizer muscles. The study by İnce and Akkuş [39] on elite weightlifters revealed that elite weightlifters developed postural deviations like forward head posture, curved shoulders, and increased lumbar lordosis due to repetitive high-intensity exercises that stress particular groups of muscles. Postural changes resulting from muscle imbalances between the anterior and posterior muscle groups were identified as major factors that raised the possibility of acute and overuse injuries.

Several studies have emphasized the biomechanical stresses of weightlifting by highlighting the connection between postural problems and fatigue-related muscle imbalances [40,41]. These studies emphasized that weightlifters who perform repetitive high-intensity movements overuse certain muscle groups, generating imbalances that impair posture and elevate their chances of injuries. According to Abdelraouf et al. [42] weightlifting techniques frequently produce muscular imbalances in the shoulder area because athletes develop strong internal rotators and adductors. In contrast, their external rotators and abductors remain underdeveloped. Weightlifting mechanics drive these imbalances by emphasizing pushing and pulling actions that strengthen particular muscles while ignoring others [43].

The results demonstrated significant increases in scapular balance angle and lateral scapular slide distance following HL-F training, which resulted in a more protracted scapular position and shoulder girdle asymmetry (scapular dyskinesis). Previous research has established that excessive muscle fatigue leads to poor scapular stability, which could lead to musculoskeletal dysfunction and postural problems. Sciascia and Kibler [44] found that muscle fatigue or weakness in the serratus anterior and lower trapezius stabilizers results in scapular dyskinesis, which creates postural imbalances through rounded shoulders and forward head posture. A study by Moon and Kim [45] revealed that this condition can further contribute to shoulder dysfunction and neck pain due to mechanical issues between the neck and scapula.

Although proprioceptive measures were not included in our protocol, the observed postural breakdown could also be explained by muscle fatigue from HL-F, which compromises joint stability and movement control, as supported by prior studies documenting fatigue-related proprioceptive deficits. The research demonstrates that fatigue creates inaccuracy in perceiving joint position sense, which leads to increased risks of injuries and dysfunctions through impaired neuromuscular control and reduced feedback from fatigued muscles, affecting primarily athletes who perform repetitive overhead movements [46]. Additionally, a more recent review by Panagiotopoulos and Crowther [47] highlighted that scapular dyskinesis, often caused by muscular fatigue, leads to poor posture and a greater risk of shoulder injuries.

### 4.2. Effect of the HL-F on Core Endurance

The prone plank endurance test revealed a significant reduction in core endurance following HL-F training as manifested by decreased holding time. This decline can be attributed to the cumulative fatigue experienced by the stabilizing musculature, particularly the rectus abdominis, transverse abdominis, and oblique muscles. Core endurance is essential for maintaining proper posture and executing weightlifting with efficient movement patterns. Impairment of core endurance may increase susceptibility to movement compensation. A strong core stabilizes the spine, enhances balance, and ensures correct body alignment, reducing the likelihood of injuries during physical activities [48].

At the neuromuscular level, core muscles engage through a feed-forward activation pattern during upper limb movements, supporting the efficient transfer of power from the core and promoting coordinated, dynamic motion of the limbs [49,50]. To further support this concept, Vasseljen et al. [51] reported that trunk stabilizers function as a feed-forward system, contracting before extremity movement to enhance overall stability. Consequently, optimal shoulder mobility and stability may depend on the activation of core muscles, highlighting their interconnected role in upper-extremity performance.

In their 2021 study, Lyu et al. investigated the effects of local and general fatiguing exercises on both disturbed and static postural control. They reported that both types of exercise significantly impaired trunk stability, with local fatigue having a more pronounced effect. The study emphasized the importance of considering the kind of fatigue in activities requiring precise balance and postural control [52]. Exhaustive local exercises impair postural control when they cause a strength loss of at least 25–30% of maximal voluntary contraction. Fatigue alters the sensory inputs and motor outputs involved in maintaining posture, prompting compensatory strategies to mitigate balance disturbances [53]. Paillard [54] proposed conceptual models to clarify how the central nervous system prioritizes specific sensory information and adapts motor strategies in response to muscle fatigue.

### 4.3. Comparison of the HL-F and HL-NF Conditions

No significant differences existed between HL-F and HL-NF baseline (unloading) measurements (*p* > 0.05), while HL-F produced significant post-exercise deteriorations, unlike HL-NF. This suggests that training that does not reach failure preserves better postural control and muscular endurance. This finding lends credence to the notion that training to non-failure may be a more sustainable approach for strength and conditioning without compromising biomechanical alignment [18].

There is controversy over the effects of resistance training to failure, with some studies showing that training to failure results in greater strength and hypertrophy [55,56], while others have found that training to failure and near failure yield similar results [13,57]. Research has also shown that training to failure may be detrimental [56,58]. Suchomel et al. [59] pointed out that training to failure is unnecessary to maximize muscle strength, and that a combination of heavy and light loads can increase strength. Hartmann et al. [60] stated that a high-load (80% 1RM) strength-power session conducted 1–2 days before competition would yield better maximum strength, peak power, and impulse size than training at 30% 1RM. This has made it difficult to establish clear training guidelines, which call for individualized program design based on the athlete’s expertise and training goals.

This study used a within-subject design to reduce biological variation and control for genetic variation, diet, training experience, and sleep [56,57,58,59]. The comparison of resistance training to failure (RT-F) and resistance training not to failure (RT-NF) within the same individuals reduced confounding variables. It allowed for a more precise examination of the adaptations to each protocol [14].

The results of this study are essential for developing weightlifting programs for young recreational weightlifters. Coaches and clinicians may wish to incorporate non-failure training methods to maintain postural stability and core function. Acute fatigue levels may be monitored, and immediate recovery strategies implemented to mitigate the adverse effects of failure-based training. Furthermore, individualized programming that accounts for load, volume, and recovery may enhance long-term musculoskeletal health and performance among weightlifters.

### 4.4. Limitations

This study has limitations that should be considered when interpreting and generalizing the findings. First, only high-load training was examined, which may not fully represent the effects of training to failure with lower loads. Training to muscle failure becomes increasingly critical when using lower loads (e.g., 30% of maximal force) because of the delayed recruitment of larger motor units [61]. Future studies should explore whether similar adaptations occur across different loading conditions. Second, the postural assessment was restricted to the upper body, limiting the applicability of the findings to whole-body postural control, particularly the lower quarter. Since postural control plays a role in maintaining stability under heavy loads, further research should evaluate how high-load failure (HL-F) training influences center-of-gravity perturbations and postural adjustments across the entire body, integrated with electromyographic data to confirm muscular fatigue. Third, Sex × condition interactions were not analyzed as this was beyond the study’s primary scope; future research should examine potential sex-specific responses to training failure, particularly given physiological differences in fatigue resistance between males and females. Finally, this study focused exclusively on weightlifters, without considering powerlifters. Recent research suggests that powerlifters and weightlifters adopt different postural control strategies based on load intensity, which may limit the generalizability of these findings [62].

## 5. Conclusions

High-load resistance training to failure elicits greater acute postural deviations and reductions in core endurance than non-failure training. These changes appear to reflect transient fatigue-related effects rather than long-term adaptations. Non-failure training may therefore represent a more sustainable strategy for maintaining postural control during high-load upper-extremity exercises in recreational lifters, with direct implications for reducing acute injury risk while optimizing lifting performance and movement quality.

## Figures and Tables

**Table 1:** The values of the postural angles and the core endurance plank test.

Variables	Testing Conditions	Unloading Condition (Mean ± SD)	Load Condition (Mean ± SD)	*p* Value	MD (95% CI)
CVA	HL-F	49.04 ± 6.73	44.91 ± 5.16	0.003 *	−4.13 (−6.79 to −1.46)
	HL-NF	50.16 ± 7.68	48.53 ± 5.96	0.341	−1.47 (−4.53 to 1.59)
	*p* value	0.459	0.005 *		
	MD (95% CI)	1.12 (−2.09 to 4.33)	3.62 (1.13 to 6.10)		
Thoracic kyphosis angle	HL-F	38.43 ± 7.28	51.72 ± 11.25	<0.001 *	13.29 (7.90 to 18.67)
	HL-NF	40.75 ± 8.25	44.62 ± 9.73	0.162	3.68 (−8.1792 to 0.8192)
	*p* value	0.236	0.021 *		
	MD (95% CI)	2.320 (−1.53 to 6.17)	7.10 (1.16 to 13.08)		
Scapular balance angle	HL-F	4.57 ± 0.92	7.53 ± 1.01	<0.001 *	2.96 (2.46 to 3.54)
	HL-NF	4.72 ± 1.1	4.98 ± 1.06	0.068	0.41 (−0.03 to 0.85)
	*p* value	0.510	<0.001 *		
	MD (95% CI)	0.15 (0.31 to 0.60)	2.55 (2.03 to 3.16)		
Lateral scapular slide distance	HL-F	0.98 ± 0.17	1.7 ± 0.23	<0.001 *	0.72 (0.81 to 0.63)
	HL-NF	0.99 ± 0.21	1.1 ± 0.19	0.164	0.11 (0.02 to 0.19)
	*p* value	0.815	<0.001 *		
	MD (95% CI)	0.01 (0.09 to 0.07)	0.7 (0.50 to 0.81)		
Prone plank time	HL-F	85.38 ± 9.47	78.54 ± 8.92	0.002 *	−6.84 (−10.93 to −2.74)
	HL-NF	86.29 ± 8.07	83.65 ± 9.47	0.182	−2.64 (−6.55 to 1.27)
	*p* value	0.645	0.015 *		
	MD (95% CI)	−0.91 (−4.82 to 3.01)	5.11 (1.01 to 9.20)		

* *p* value less than 0.05 means significant difference, CVA; Craniovertebral angle, HL-F; high load to failure, HL-NF; high load to non-failure.

## Data Availability

The data are available from the corresponding author upon reasonable request.

## Abbreviations

The following abbreviations are used in this manuscript:

HL-F	high-load to failure
HL-NF	high-load to non-failure
CVA	craniovertebral angle
1RM	one-repetition maximum
SPSS	Statistical Package for Social Sciences
SD	standard deviation
CI	confidence interval
